# Photobiomodulation in the treatment of xerostomia associated with hyposalivation in a pediatric patient with systemic scleroderma

**DOI:** 10.4322/acr.2020.220

**Published:** 2020-12-14

**Authors:** Analú Barros de Oliveira, Túlio Morandin Ferrisse, Karina Borges Salomão, Marina Lins Miranda, Andreia Bufalino, Fernanda Lourenção Brighenti

**Affiliations:** 1 Universidade Estadual de São Paulo (Unesp), Faculdade de Odontologia de Araraquara, Departamento de Morfologia e Clínica Infantil, Araraquara, SP, Brasil; 2 Universidade Estadual de São Paulo (Unesp), Faculdade de Odontologia de Araraquara, Departamento de Diagnóstico e Cirurgia, Araraquara, SP, Brasil

**Keywords:** scleroderma, systemic, low-level light therapy, pediatrics, laser therapy, xerostomia

## Abstract

Scleroderma is a rare autoimmune disease characterized by excessive collagen production. The oral manifestations of the patient with scleroderma can include microstomia, xerostomia, and changes in the resorption teeth. We report the case of a 7-year-old female patient diagnosed with systemic scleroderma where photobiomodulation therapy was used to treat xerostomia associated with hyposalivation. She attended a pediatric clinic and presented with dry and rigid facial skin, trismus, xerostomia, malocclusion, and difficulty swallowing. Stimulated salivary flow was assessed before, during, and after treatment. Photobiomodulation therapy was conducted at four points at the sublingual glands with 660 nm, 100 mW, and 0.8 J/cm^2^ to each point; eight points at the parotid glands; and six points at the submandibular glands with 808 nm, 100 mW, and 0.8 J/cm^2^ for 8 seconds at each point. After this therapy, an increase in salivary flow, remission of the xerostomia, and an improvement in mastication and swallowing were observed. Photobiomodulation therapy was effective in controlling xerostomia in this pediatric patient, resulting in increased salivary flow and an improvement in her quality of life.

## INTRODUCTION

Scleroderma (Sc) is a rare autoimmune disease that causes excessive production of collagen, leading to hardening of the skin. It affects the joints, muscles, blood vessels, and some internal organs such as the lungs and heart. Until now the etiology and pathogenesis of Sc are poorly understood, possibly due to the rarity of this disease.^1^

Sc is divided into two types, systemic (SSc) or localized (LSc).^2^ LSc is the most common form of Sc; it occurs in about 90% of cases and classically presents a benign and self-limiting evolution, and is confined to the skin and/or underlying tissues. SSc is the rarest and severe form of Sc, affecting skin and some internal organs.^3^ Sc presents slow, progressive, and incapacitating evolution, but it can also occur rapidly and lead to death due to the involvement of the internal organs.^1^

According to the literature, Sc can manifest at any age, but it is more commonly reported in women over 30 years old.^1^ Herrick et al.^4^ evaluated the annual incidence of SSc in childhood in the United Kingdom, and concluded that involvement in children under 16 years old is extremely rare.

Oral manifestations may include tongue stiffness, microstomia, and hyposalivation followed by xerostomia and different degrees of bone and teeth resorption.^5^ ^,^ ^6^ Non-stimulated salivary flow is considered to be hyposalivation with values lower than 0.29 mL/min and 1.0 mL/min for stimulated salivary flow.^7^ ^,^ ^8^ Hyposalivation can lead to poor oral health and cause adverse impacts on daily life, such as discomfort and pain. Thus, the aim of this article is to report successful treatment with photobiomodulation therapy (PBMt) for xerostomia followed by hyposalivation in a pediatric patient diagnosed with Sc.

## CASE REPORT

A 7-year-old girl was attended the emergency room and the clinical examination showed dry and rigid facial skin, trismus, xerostomia, malocclusion, and several carious lesions involving dental elements 54, 55, 64, 74, 75, 84, and 85 (Figures 11B).

**Figure 1:**
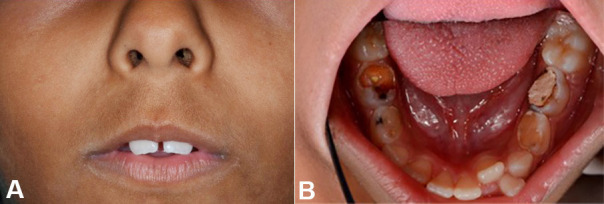
**A –** Clinical aspects presented by the patient. It was observed dry skin and microstomia on the extraoral exam; **B –** Several carious lesions and dental crowding in the intraoral exam.

Dental treatment was started to adjust the oral environment; xerostomia and hyposalivation were treated with PBMt. Photon Lase III equipment (PL7336, DMC, São Carlos, São Paulo, Brazil) was used for the treatment and was adjusted to red laser (660 nm—InGaAlP), power of 100 mW, with an energy density of 0.8 J/cm^2^, 8 seconds per point in the sublingual glands. For the parotid and submandibular glands the equipment was adjusted for infrared laser (880nm—AsGaAl) power of 100 mW, with an energy density of 0.8 J/cm^2^, 8 seconds per point. The application method was punctually located, with 1 cm distance between the application points (8 points in the parotid glands, 6 points in the submandibular glands, and 4 intra-oral points at sublingual glands), totaling six sessions of laser application with a 72-hour interval between sessions.

After three sessions of PBMt, lubrication of the oral mucosa and remission of the symptomatology were reported by the patient. Additionally, initial stimulated salivary flow was less than 0.1 mL per minute, and after six laser sessions an increase in salivary flow of 1.0 mL per minute was observed (Figures 22B).

**Figure 2:**
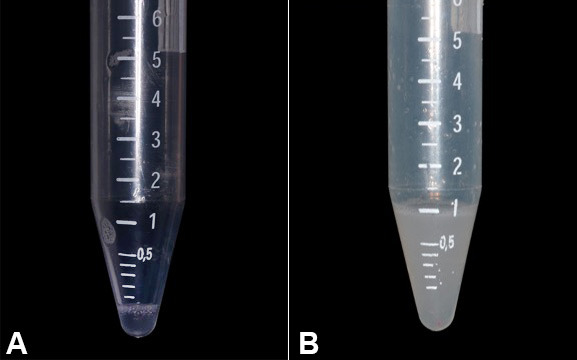
**A –** Salivary flow stimulated before the PBMt treatment (salivary flow rate <0.1 mL/min); **B –** After six sessions of PBMt there were remission of the symptomatology and an increase in the salivary flow rate to 1.0 mL/min were observed.

During the period of time that the photobiomodulation therapy was applied the patient did not receive any systemic or topic adjunct treatment.

To date, the patient has had 2 years follow-up without change in the clinical aspects obtained with the proposed therapy. Furthermore, the patient reports an improvement in mastication and swallowing.

## DISCUSSION

Frequently, the correct diagnosis of SSc is difficult to obtain, because the early stage of the disease is very similar to other connective tissue diseases, such as systemic lupus erythematosus, polymyositis, and rheumatoid arthritis.^3^ In the present case the patient was already diagnosed with Sc. However, dental clinics should be aware of the oral manifestations of Sc in order to help the diagnosis and treatment management.

Few studies in the literature have evaluated the effect of PBMt in salivary glands to improve salivary flow however outcomes have been controversial.^9^ ^-^ ^11^ Fidelix et al.^9^ performed a randomized clinical trial in xerostomia associated with primary Sjögren syndrome. The study group received PBMt twice a week for 6 weeks. The laser irradiation was adjusted to 808 nm, 100 mW, 4.0 J/cm^2^ per irradiation point. The researchers did not find any differences related to xerostomia and salivary flow rate. The Biswas et al.^10^ study evaluated the effect of PBMt in the frequency of major water channel protein aquaporin 5 (AQP5) in salivary glands during hyperglycemia in mice models. The researchers found an improvement in salivary function by increasing AQP5 membrane distribution in mice that received PBMt. Moreover, Lončar et al.^11^ found a significance difference in salivary flow between PBMt groups when compared with the control group in an elderly population. The researchers concluded that the effects of PBMt on salivary glands are not only stimulating the salivary flow but also regenerating the quality of the saliva.

However, further studies to confirm the potential of using PBMt to treat xerostomia and hyposalivation symptoms should be carried out. Additionally, the precise action mechanism of PBMt in xerostomia and hyposalivation is still unknown and poorly understood, and should be evaluated in the future. There are no precise protocols in the literature regarding PBMt for the treatment of hyposalivation or xerostomia. Basically, the protocols differ by the wavelength used.^12^

The treatment of xerostomia followed by hyposalivation is wide and normally involves the use of systematic sialagogues, such as pilocarpine and cevimeline.^13^ However, these approaches have many side effects. Other options can be the use of intraoral topical agents, such as chewing gums, salivary stimulants, and salivary substitutes, plus other interventions, such as acupuncture and electrostimulation, but with questionable results.^13^ Thus, due to the capability of the PBMt in recovering the quality of saliva and being easy to apply without side effects, these treatments have interest points that must be confirmed with further studies.

The present case describes the first case in the literature that evaluated the effect of PBMt on xerostomia in a pediatric patient diagnosed with SSc. Our results strengthen the importance of PBMt as the treatment for xerostomia associated with hyposalivation in SSc patients, since hyposalivation is a common oral manifestation in Sc patients, affecting 52.5% of these patients.^14^ Saliva plays an important role in mastication, speech, swallowing, gustatory sensitivity, protection of the oral mucosa, and formation of the food bolus. Also, it has antibacterial, antifungal, antiviral activities, and participates in the mineral balance of dental enamel. In this context, deaths from SSc at a ratio of 4% have been reported and it is likely that this is associated with hyposalivation and its adverse impacts on bad nutrition.^15^ Thus, any effort to treat and recover the salivary flow rate and the quality of saliva is very significant.^16^

With this report we have recommended the use of PBMt as a possible treatment of xerostomia associated with hyposalivation in pediatric SSc patients considering its notable success in salivary flow. However, further studies in the form of randomized, controlled, clinical trials with adequate samples are necessary to confirm the findings of this case report, and in vitro studies are necessary to evaluate the exact mechanism involved in PBMt and salivary glands.

## Abbreviations

**LLLT**: low-level laser therapy, **LSc**: localized scleroderma, **mW**: miliwatts, **nm**: nanometer, **Sc**: scleroderma, **SSc**: systemic scleroderma
